# Coping with Stress and the Emergence of Multidrug Resistance in Fungi

**DOI:** 10.1371/journal.ppat.1004668

**Published:** 2015-03-19

**Authors:** Erika Shor, David S. Perlin

**Affiliations:** Public Health Research Institute, Rutgers Biomedical and Health Sciences, Newark, New Jersey, United States of America; Duke University Medical Center, UNITED STATES

The incidence of invasive fungal infections poses a serious global health threat, killing nearly 1.4 million people a year—a number comparable to deaths from tuberculosis. Factors that contribute to this disease burden and high mortality include insufficient diagnostics and treatment, and the increasing numbers of individuals with immune system defects who are particularly susceptible to fungal pathogens [1].

Because of the high risk of fungal infections in immunocompromised individuals—e.g., low–birth-weight neonates or patients undergoing organ transplantation or chemotherapy—it has become common medical practice to utilize antifungal prophylaxis in these patients during immunosuppression [2–4]. However, the expanding use of antifungal drugs has been associated with increasing incidence of antifungal drug resistance resulting from inherently less sensitive species and/or acquisition of drug class–specific resistance mechanisms [5]. Most alarming in recent years, multidrug-resistant strains of certain *Candida* species have emerged that are resistant to azoles and echinocandins, the two most widely used classes of antifungal drugs [6,7].

In this article we explore the notion that frequent and prolonged exposures of fungal cells to antifungal drugs activate fungal stress responses, which both support the short-term cellular adaptation to the drugs [8,9] and promote genetic instability to facilitate the emergence of stable drug resistant mutants refractory to therapy [9,10], including multidrug-resistant (MDR) strains (Fig. 1). Antifungal drug-induced stress has been associated with genetic instability in such distantly related fungi as *Candida* and *Cryptococcus* [11,12], suggesting that it is a broadly conserved phenomenon. In this article, we focus on drug resistance in *Candida* spp. because of its clinical significance, especially with the development of emerging multidrug resistance, and because mechanisms of drug resistance and stress-associated genetic instability are best studied in *Candida*, both in the clinic and in the lab.

**Fig 1:**
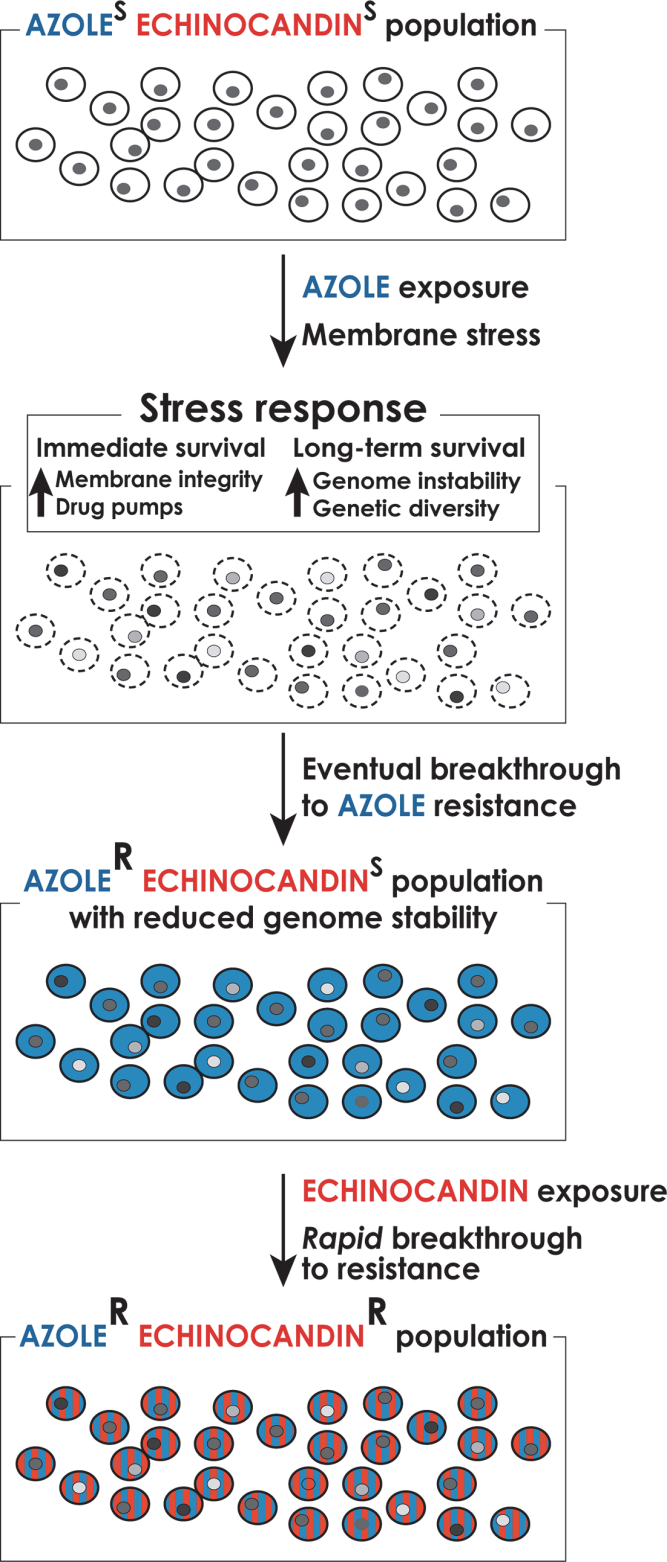
A model for the formation of multidrug-resistant strains of *C*. *glabrata*. Long-term exposure to antifungal drugs triggers stress responses that both help maintain cellular integrity in the face of acute stress and promote genomic instability and genetic diversity to facilitate the emergence of genetic variants with enhanced intrinsic resistance. In the case of long-term prophylaxis with azole class drugs in *Candida glabrata*, this process results in formation of azole-resistant mutants with reduced genome stability. These mutants, when challenged with echinocandin class drugs, rapidly develop echinocandin resistance as well, resulting in formation of multidrug-resistant *C*. *glabrata*.

## Genetic Basis of Antifungal Drug Resistance

The most prevalent fungal pathogens belong to the *Candida* genus, with *C*. *albicans* and *C*. *glabrata* being the most common and second or third most commonly isolated bloodstream fungal pathogens in the United States [13,14], respectively. For more than three decades, *Candida* infections have been treated with azole antifungal agents, which target fungal membranes by inhibiting the biosynthesis of ergosterol. However, nearly 20% of *C*. *glabrata* strains exhibit intrinsic resistance to azoles, and even susceptible strains rapidly acquire resistance. This inherent resistant property has contributed to *C*. *glabrata* being the most frequently isolated *Candida* species in some high-risk centers [15,16]. To address this issue, echinocandin drugs are now recommended as first-line therapy to treat a range of candidiasis [17], since they do not exhibit cross-resistance to azole-resistant yeasts. A relatively recent addition to the antifungal drug repertoire, echinocandin class compounds target the fungal cell wall by inhibiting the biosynthesis of the key cell wall polymer β-(1,3)D-glucan. Troublingly, however, *C*. *glabrata* resistance to echinocandins is on the rise [18], with >10% of isolates now showing resistance in some hospital settings [7]. Many of the echinocandin-resistant *C*. *glabrata* isolates are also resistant to azoles, leaving extremely few options to treat patients infected with MDR strains [7].

Clinical resistance to echinocandins resulting in treatment failures is nearly always due to limited mutations in two highly conserved “hot spot regions” of genes encoding subunits of β-glucan synthase: *FKS1* for most *Candida* species and *FKS1* and/or *FKS2* in *C*. *glabrata* [19,20]. Echinocandin resistance is always acquired during therapy, and the resulting amino acid changes in glucan synthase significantly decrease the sensitivity of the enzyme to the drug, resulting in higher MIC values and reduced pharmacodynamic responses [20].

In contrast to the echinocandins, azole drug resistance resulting in clinical failure may be caused by a variety of genetic changes, most of which affect the expression of fungal drug transporters or the structure and/or expression of fungal drug targets [9]. For instance, resistance to azoles can be caused by overexpression of transporters that mediate drug efflux out of the cell [21]. This overexpression can be achieved by amplification of the transporter genes, by large scale chromosomal rearrangement or chromosome gain that result in higher transporter gene copy number, or by point mutations in transcription factors that regulate transporter gene expression [22]. Resistance to azoles can also be mediated by mutations in the gene encoding their cellular target—the Erg11 protein, or by mutations in transcriptional regulators of *ERG11* that result in Erg1 overexpression [23,24]. In *C*. *albicans*, clinical resistance to azoles frequently occurs in a stepwise manner, with a gradual increase in resistance associated with a sequential accumulation of mutations and genome rearrangements that together result in high-level azole resistance [25]. In contrast, in *C*. *glabrata* a single gain-of-function mutation in a transcriptional activator of drug efflux pumps is often sufficient to confer high-level azole resistance [26].

## Theoretical and Experimental Support for Stress-Induced Genetic Instability

How do genetic changes that cause drug resistance arise and become fixed in fungal populations? The classical theory of evolution and natural selection posits that rare, random mutations arise in a population (e.g., due to occasional spontaneous errors in DNA replication and repair processes) and that a change in conditions, such as appearance of antifungal drug, would favor mutants that happen to be more fit under the new conditions (i.e., resistant to the drug). However, it seems clear that in thriving populations well adapted to their stable environments, genetic diversity is far less valuable than in maladapted populations, or during times of environmental change. From this point of view, a winning evolutionary strategy for an organism would contain the capacity to enhance its genetic diversity specifically during the times of stress. Indeed, mathematical modeling shows that the ability to generate increased genetic diversity by mutation or recombination specifically during the times of decreased fitness, i.e., stress, is itself a beneficial trait that would be favored by natural selection [27,28]. In other words, an organism with the capacity to modulate its spontaneous mutation and recombination rates—keeping them low during conditions of low stress and increasing them during conditions of high stress—would have a selective advantage over organisms with constant (either constitutively low or high) mutation and recombination rates.

The modeling studies are supported by empirical observations of increased genetic instability in fungi exposed to stresses such as high temperature, starvation, proteotoxic stress, and antifungal drugs. Starving laboratory strains of baker’s yeast (*Saccharomyces cerevisiae*) induces gross chromosomal rearrangements at rates several-fold higher than non-starving cells [29]. Interestingly, only a fraction of these rearrangements shows increased fitness relative to the parent strain under the starvation conditions, suggesting that rearrangements arise not by the selection of pre-existing genetic variants but by random starvation-induced genetic variability [29]. Furthermore, growth of laboratory yeast strains at high temperature or in the presence of antifungal drugs strongly induces gross chromosomal rearrangements and chromosomal gain or loss [30]. Studies of clinical *C*. *albicans* isolates from patients treated with azoles show that these strains frequently exhibit gross chromosomal rearrangements and/or aneuploidy [11]. Likewise, in *C*. *glabrata* isolated from patients, chromosomes are frequently reshuffled resulting in new genetic configurations, including appearance of small chromosomes [31,32]. Moreover, passaging *C*. *albicans* through a mouse in the absence of drug treatment increases chromosomal rearrangements relative to *C*. *albicans* passaged in regular laboratory medium, indicating that the stresses encountered in vivo, such as oxidative stress and elevated temperature promote genetic instability [33].

## Roles of Cellular Stress Responses in Genetic Instability and Drug Resistance

Cellular stress responses are generally considered in terms of their role in helping cells tolerate and survive acute stress. For instance, echinocandin exposure compromises the cell wall and induces a number of stress signaling cascades, including those dependent on protein kinase C (PKC), Ca^2+^/calcineurin, HSP90 and high osmolarity glycerol (HOG) kinase, and up-regulation of chitin production to enhance cell wall stability [8,20]. These compensatory mechanisms produce a drug tolerant state where cells appear to resist the fungicidal properties of echinocandin drugs. The enhanced drug tolerance may explain why significant levels of surviving persister *C*. *albicans* cells were observed in mice after echinocandin treatment [34]. It is likely that stress response–mediated enhanced drug tolerance allows some cells sufficient time to develop a resistance-conferring mutation in *FKS1* or *FKS2*.

However, the observations of chromosomal instability during stress strongly suggest that another role of cellular stress responses is to help increase genetic diversity during stress, presumably by altering the machinery responsible for maintaining genome integrity [10]. Indeed, several fungal stress response factors have been implicated in promoting genetic instability during stress. For instance, in *S*. *cerevisiae*, general stress response transcription factors Msn2 and Msn4 help promote certain types of mutagenesis in response to proteotoxic stress [35]. Furthermore, in both *Saccharomyces* and *Candida*, stress-induced aneuploidy depends on the function of a stress-inducible protein chaperone HSP90, which regulates the folding of a number of client proteins that function in chromosome segregation and cell cycle progression [30,36]. Another mechanism of stress-induced aneuploidy has been observed in several *Candida* species, including *C*. *albicans* and *C*. *glabrata*, involving mis-regulation of the fungal cell cycle shortly upon exposure to an antifungal agent. Within a few hours of exposure to azoles or echinocandins, *Candida* cells were observed to uncouple spindle formation and nuclear division from cell division, ultimately leading to the formation of tetraploid cells that underwent unequal division to produce aneuploid progeny [37].

While induction of aneuploidy appears to be an early event following stress, aneuploidy can promote other types of genomic rearrangements and mutagenic lesions [38]. This effect of aneuploidy is attributed to the fact that it alters gene dosage of a subset of the genome, thus disrupting the stoichiometry of complexes involved in chromosome maintenance and DNA repair whose components are encoded by different chromosomes.

## Clinical Implications of Stress-Induced Genetic Instability

The potential for stress-induced genetic instability is especially troubling for drugs such as azoles, which are fungistatic—i.e., they do not kill fungal cells but only arrest their growth. As outlined above, arrested cells activate stress responses that promote genome destabilization and genetic diversity. Indeed, data from the clinic indicating rapid development of drug resistance following initiation of therapy [39] support the notion that exposure of *C*. *glabrata* to an azole may create fungal cells with highly variable, “evolvable” genomes capable of quick breakthrough of resistance to any other types of drug, such as echinocandins (Fig. 1). Thus, patients encountering fluconazole prophylaxis may show infections with azole resistant *C*. *glabrata* followed by rapid breakthrough on echinocandin therapy, resulting in multidrug-resistant strains. In light of this phenomenon, it may be necessary to review the current practice of prophylaxis in some settings, which promotes such genetic instability. Furthermore, there is an urgent need for new fungicidal drugs, for safe combinations of antifungal drugs that would require mutagenesis of more than one target to create resistance (a much more rare event), and for a deeper understanding of mechanisms underlying stress-induced genetic instability so that they could potentially be pharmacologically mitigated during antifungal therapy.
